# Highly efficient production of HIV-1_AD8_ gp120 in mammalian cells

**DOI:** 10.1128/jvi.01359-25

**Published:** 2025-11-07

**Authors:** Tanvi Mathur, Shamim Ahmed, Durgadevi Parthasarathy, Alon Herschhorn

**Affiliations:** 1 Division of Infectious Diseases and International Medicine, Department of Medicine, University of Minnesota 199672, Minneapolis, Minnesota, USA; 2 The Molecular Pharmacology and Therapeutics Graduate Program, University of Minnesota 5635, Minneapolis, Minnesota, USA; 3 The College of Veterinary Medicine Graduate Program, University of Minnesota 5635, Minneapolis, Minnesota, USA; 4 Institute for Molecular Virology, University of Minnesota 5635, Minneapolis, Minnesota, USA; 5 Microbiology, Immunology, and Cancer Biology Graduate Program, University of Minnesota 5635, Minneapolis, Minnesota, USA; Icahn School of Medicine at Mount Sinai, New York, New York, USA

**Keywords:** envelope glycoproteins, HIV-1, vaccine

## Abstract

Human immunodeficiency virus type 1 (HIV-1) envelope glycoproteins (Envs) interact with the CD4 receptor and CCR5/CXCR4 coreceptor expressed on target cells to mediate viral entry. Infection is initiated when the HIV-1 gp120 subunit of Envs binds to host receptors. gp120 can also be expressed as a soluble protein and is routinely used for different biophysical and immunization studies. Here, we compared the transient and stable expression of monomeric HIV-1_AD8_ gp120 in two closely related 293 cell lines grown in suspension. We used an HIV-1_AD8_ gp120-expressing plasmid to generate 293 cell lines that stably expressed HIV-1_AD8_ gp120 under selection of puromycin. In parallel, we used polyethyleneimine (PEI) to transiently transfect FreeStyle 293 F cells with the identical HIV-1_AD8_ gp120-expressing plasmid. Stable 293-AD8gp120 cells grew in media complemented with a feed supplement to a density of >1E7 cells/mL and produced an average of ~150 mg of gp120/liter of the culture, which was about 50-fold higher than the yield of gp120 produced by transient transfection of FreeStyle 293 F cells. SDS-PAGE analysis of HIV-1_AD8_ gp120 produced by the two different methods showed similar patterns, but gp120 purified from the stable cell line exhibited higher homogeneity. Binding of anti-gp120 antibodies to gp120 that was produced by the two methods was comparable according to ELISA. Both gp120s could bind equally well to soluble CD4 and compete with infection of viruses pseudotyped with HIV-1_AD8_ Envs. Our findings highlight multiple advantages of producing HIV-1_AD8_ gp120 by stable expression for downstream applications.

**IMPORTANCE:**

There are approximately 40.8 million people living with HIV-1 (PLWH) worldwide, with an estimate of about 1.3 million new HIV-1 infections in 2024, highlighting the urgent need for an effective HIV-1 vaccine and cure strategies. Here, we describe a highly efficient method to produce soluble HIV-1 gp120, which is intensively used in viral assays and for vaccine development. The method may be helpful in research work that needs high amounts of proteins for diverse experiments.

## INTRODUCTION

The human immunodeficiency virus type 1 (HIV-1) pandemic continues to spread, with 1.3 million (1.0–1.7 million) people who acquired HIV-1 and 630,000 (490,000–820,000) people who died from HIV-1-related causes globally in 2024 (https://www.who.int). FDA-approved antiretroviral therapy (ART) includes inhibitors of HIV-1 reverse transcriptase (1–3), integrase (4, 5), protease (6), envelope glycoproteins (Envs) (7–11), and drugs that target HIV-1 capsid (12–14) and the cellular host receptors CD4 and CCR5 (15, 16). Although very effective, ART does not cure HIV-1 in people living with HIV-1 (PLWH), and upon ART discontinuation, HIV-1 replenishes the viral population from latent reservoirs and/or from low levels of replicating viruses (17–20). Thus, new approaches at different levels of development are and have been constantly tested for targeting HIV-1 persistence (21–28).

HIV-1 Envs mediate viral entry, have a key role in critical virus-host interactions, and are a target of intensive efforts to develop an HIV-1 vaccine (29–39). On the surface of HIV-1 virions, the Envs are assembled into a trimeric spike consisting of three gp120 exterior subunits and three gp41 transmembrane subunits (40–42). The gp120 subunit interacts with the host CD4 receptor and CCR5/CXCR4 coreceptors, while the gp41 subunit facilitates membrane fusion (43–48). Receptor binding is associated with extensive structural rearrangements that result in Env conformational transitions from a closed conformation (State 1) to downstream states (State 2 and State 3) (49–52). Env State 1 is a metastable conformation that contributes to low Env presentation on virions or infected cells and thus poses a significant challenge to study Envs in their native state. Notably, the combination of high Env robustness (53), which allows HIV-1 Envs to tolerate amino acid changes while maintaining entry competency; heavy glycan shield (54–58), which masks critical and vulnerable Env sites; Env conformational flexibility (59–61); and HIV-1 cell-to-cell transmission (62–65) facilitates the exceptional ability of HIV-1 Envs to escape broadly neutralizing antibodies (66–72).

Studies of HIV-1 Env biology and designing HIV-1 vaccine candidates typically require large amounts of soluble HIV-1 gp120. In general, bacterial expression systems allow high protein yield, but production of active proteins can be challenging when the native proteins rely on post-translational modifications, i.e., glycosylation, or specific chaperones for function. Mammalian cells have become a popular choice for recombinant protein production due to their ability to express proteins with native-like post-translational modifications (PTMs) (73). Commonly used cells for recombinant protein production include human embryonic kidney (HEK293) and Chinese hamster ovary (CHO), along with their various derivatives. But other cell lines, such as Sf9, HKB11, and CAP-T, have been used as well (74). Recombinant proteins can be expressed by transient or stable transfection, and there are several advantages and disadvantages for each method. Transient transfection offers speed and flexibility but requires a relatively large amount of DNA. Due to the complexity of some proteins, it is sometimes difficult to produce significant amounts of specific proteins that could be effectively used for downstream applications. On the other hand, the development of stable cell lines is a long and time-consuming process but remains essential for applications requiring long-term, high-level protein expression and ease of upscaling. Protein expression using stable cell lines can significantly increase protein yields, and the process is reproducible, but long-term stability of the clones depends on multiple factors including the cells, specific gene of interest (GOI), protein expressed, and the sites of GOI integration into the cellular genome. Nevertheless, stable gene expression requires low amounts of plasmid DNA and can usually be scaled up for large production of proteins (75). CHO cells are genetically stable and can be transfected, enabling both transient and stable expression workflows (76–78). However, one of their limitations is nonhuman PTMs such as galactose-α1,3-galactose and N-glycolylneuraminic acid, which may be immunogenic in humans (79). Alternatively, HEK293 cells are often used; HEK293 cells can perform efficient γ-carboxylation, a critical modification for function of some proteins (79, 80). Similar to CHO cells, HEK293 cells are well-suited for culturing in suspension and support both transient and stable expressions of recombinant proteins. Over the years, several derivatives of the HEK293 lineage have been developed to enhance specific characteristics. One such variant, HEK293S GnTI^-^, has been adapted for growth in suspension (77). The optimal balance between growth and production is an important factor to consider when growing mammalian cells for either transient or stable protein expression. Limits on cell growth could significantly reduce the yield of the protein being produced. Here, we generated stable adherent HEK293 cells that express soluble HIV-1 gp120 and adapted the cells to grow in suspension. Growth in a BalanCD HEK293 medium enabled high-yield recombinant protein expression of ~150 mg/liter using a batch-feed scheme, which is designed to boost protein production by increasing viable cell density and prolonging culture duration. We compared the protein production, antigenicity, and expression patterns of gp120 purified from these cells to the gp120 purified after transient expression in the commonly used FreeStyle 293 F cells.

## MATERIALS AND METHODS

### Cells

Adherent HEK293 cells were obtained from the NIH HIV reagent program, and FreeStyle 293 F cells were purchased from Gibco (ThermoFisher Scientific). Adherent HEK293 cells were maintained at 37°C and 5% CO2 in Dulbecco’s modified Eagle’s medium (DMEM) containing 10% fetal bovine serum (FBS), 100 µg/mL streptomycin, and 100 units/mL penicillin (Invitrogen, ThermoFisher Scientific). FreeStyle 293 F cells were maintained in suspension at 37°C and 8% CO2 in Freestyle expression medium (Gibco; ThermoFisher Scientific) with continuous shaking at 110–130 rpm.

### Plasmid construction

HIV-1 NL4-3 ASP Expression Vector (pcDNA3.4-based vector for expression of HIV-1 antisense protein) was obtained from the NIH HIV Reagent Program (cat# 13463). DNA sequence of the puromycin N-acetyl-transferase (puromycin resistance) gene was codon-optimized for *Homo sapiens* use, synthesized *de novo,* and cloned into the HIV-1 NL4-3 ASP vector replacing the neomycin-resistant gene. DNA sequence of gp120 followed by the DNA sequence of 3xGly and 8xHis tag was codon-optimized for *Homo sapiens* use, synthesized *de novo,* and cloned into the HIV-1 NL4-3 ASP/puromycin vector replacing the asp gene. The 1059-SOSIP expression plasmid was constructed by codon-optimizing the DNA sequence of 1059-SOSIP, *de novo* gene synthesizing, and cloning the gene into the pTwist CMV BetaGlobin WPRE Neo vector (Twist Bioscience). Gene synthesis and cloning were done by Gene Universal, Newark, DE.

### HEK293 transfection and selection

Exponentially growing HEK293 cells were used to generate stable AD8gp120-expressing cells. For transfection, 4 × 10^6^ cells were seeded in a T75 flask and incubated overnight. The next day, chloroquine (25 µM final concentration; MilliporeSigma) was added to the medium dropwise, and 5 µg of pcDNA3.4.p-AD8gp120 plasmid was transfected using calcium phosphate as previously described (81). After 12–14 hours, the medium was replaced with 12 mL fresh DMEM to reduce chloroquine toxicity. Chloroquine has been shown to significantly improve transfection efficiency and is cytotoxic to cells if not removed after up to 14 hours (82, 82–87). Cells were then selected with 0.5 µg/mL of puromycin (final concentration; MilliporeSigma), and the medium was replaced with a fresh medium containing the same puromycin concentration every few days to remove dead cells and allow selection. After ~4 weeks, cells grew with minimal cell death in the presence of 0.5 µg/mL puromycin. gp120 expression was assessed by Western blot or small-scale purification using Ni-NTA chromatography.

### HEK293 adaptation to growth in suspension

Stable HEK293-AD8gp120 cells were centrifuged, washed, and directly resuspended in BalanCD HEK293 medium (Fujifilm; Irvine Scientific) supplemented with 4 mM GlutaMAX at an initial cell density of 1 × 10^6^ cells/mL and in a total volume of 20 mL. The culture was incubated in a 37°C, 100 RPM, 8% CO2 incubator for 4–5 days, and cell density and viability were monitored daily. Adapted cells were subsequently expanded to 4 × 10^6^ cells/mL in 200 medium and then were subject to a feed schedule supplementing with 4% BalanCD HEK293 Feed (Fujifilm Irvine Scientific) every other day for ~10 days to maintain optimal growth and enhanced protein production.

### Transient transfection

FreeStyle 293 F cells or HEK293 were transfected with pcDNA3.4.p-AD8gp120 plasmid or co-transfected with pTwist 1059-SOSIP-expressing plasmid and a human furin-expressing plasmid at a ratio of 4:1 (total 1 mg DNA/1 L of cells) using PEI, as previously described (88), or Turbo (Speed BioSystems, Cat # PXX1001) according to the manufacturer’s instructions and grown for 5–10 days in a tissue culture incubator at 37°C, 8% CO_2_ with continuous shaking.

### Expression optimization

Cells in suspension. Our previous optimization studies indicated that a 1:3 ratio of DNA:PEI and transfection of 2E6 cells/mL resulted in the most efficient transfection, and we used these conditions throughout the current studies.

Adherent cells. Adherent 0.5 × 10^6^ HEK293 cells were transfected with 0.8, 2.5, or 5 µg of AD8gp120-expressing plasmid DNA and selected with 0.5 or 1 µg/mL puromycin. Expression of AD8 gp120 was assessed in HEK293 cells by small-scale Ni-NTA purification at 72 hours post-transfection and 96 hours post-transfection under puromycin selection; AD8 gp120 yields were evaluated and compared by SDS-PAGE. Based on these optimizations, 5 µg of AD8gp120 plasmid DNA was subsequently transfected into 4 × 10^6^ HEK293 cells, followed by selection with 0.5 µg/mL puromycin.

### Protein purification

Culture supernatants containing soluble gp120 or 1059-SOSIP glycoproteins were harvested from the culture of transiently transfected FreeStyle 293 F cells, HEK293 cells, or from the culture of stable HEK293-AD8gp120 cells by centrifugation at 7,000 × *g* for 1 hour and filtered using a 0.45 µm vacuum filtering system (FOXX Life Sciences, Cat# FOX-1151-RLS). The supernatant was dialyzed against Ni-NTA buffer (50 mM NaH_2_PO_4_, 300 mM NaCl; pH 7.4) overnight using a 13,000 MWCO dialysis tube (VWR), or 10× of dialysis buffer was added to the supernatant to achieve a final 1× concentration. The equilibrated supernatant was then loaded on an Ni Sepharose High Performance column (Cytiva, Cat# 29051021) connected in an AKTA Go protein purification system (Cytiva) at 4°C–8°C. The column was washed with 500 mM NaCl in phosphate buffer pH 7.4, and the protein was eluted using a linear imidazole gradient containing 50–500 mM imidazole (Millipore Sigma) in Ni-NTA buffer. In some cases, 10 mL Econo-Column chromatography columns (BioRad, Cat# 7371512) were packed with Ni-NTA agarose beads (Qiagen, Cat# 30210), and proteins were purified by gravity flow. The supernatant was pre-dialyzed against Ni-NTA buffer (50 mM NaH_2_PO_4_, 300 mM NaCl; pH 8.0), which was recommended by the manufacturer and used for all steps. The equilibrated supernatant was loaded into the column, and the beads were then washed with the Ni-NTA buffer followed by stepwise elution using imidazole (50–250 mM). Eluted fractions were analyze by 8%–16% SDS-PAGE gel (mini-PROTEAN TGX protein gels; Bio-Rad), and fractions containing the gp120 glycoprotein were concentrated followed by buffer exchange to PBS using Vivaspin 6 centrifugal concentrators (30 kDa; Cytiva, Cat# GHC-28-9323-17). Purified gp120 glycoproteins were flash-frozen and stored in aliquots at −80°C. The concentration of the protein was determined by BCA assay (Pierce, Cat# A55864).

1059-SOSIP was purified by affinity chromatography using a Galanthus Nivalis Agglutinin (GNA) pre-packed column (Sterogene Bioseparations, Cat# 937614SF4). The clarified supernatant was equilibrated with five column volumes (CVs) of PBS, loaded on the column, and the column was washed with 10 CVs of PBS at a high flow rate, followed by an additional 5 CV wash with high salt (PBS containing 500 mM NaCl, pH 7.5). The 1059-SOSIP protein was eluted with 0.1–0.3 M of methyl-α- D-mannopyranoside/PBS solution and concentrated using Vivaspin 6 centrifugal concentrators (30 kDa; Cytiva). Puriﬁed 1059-SOSIP glycoproteins were then further separated according to protein size using a HiLoad 16/600 Superdex 200 pg size-exclusion chromatography column (Cytiva).

### Deglycosylation analysis

Four micrograms of AD8gp120, purified from 293 F cells or HEK293 stable cell line, were mixed with 1 µL Glycoprotein Denaturing Buffer (10×) in 10 µL reaction volume and incubated at 100°C for 10 minutes. The reaction was then cooled down by placing the tube on ice, the tube was centrifuged for 10 seconds, and 2 µL GlycoBuffer 2 (10×), 2 µL 10% NP-40, 6 µL water, and 1 µL PNGase F (New England Biolabs) were added to the reaction tube and then incubated at 37°C for 1 hour. Deglycosylation was evaluated by SDS-PAGE analysis. Untreated AD8gp120 samples were analyzed in parallel to compare side-by-side glycosylated and deglycosylated AD8 gp120 proteins.

### Enzyme-linked immunosorbent assay (ELISA)

We used ELISA to analyze antibody binding to gp120 monomers, as previously described (89). Briefly, Immulon 2 HB 96-well plates (ThermoFisher, Cat # 3455) were coated by adding 100 µL of PBS containing 0.1 µg AD8 gp120 to each well and incubating overnight in 4°C. The next day, plates were washed three times with 0.1% Tween-20 (BioRad, Cat # 1706531) in PBS (TPBS) and blocked overnight at 4°C with 0.5% dry skim milk (BioRad, Cat # 1706404) and 0.1% Tween-20 in PBS (MTPBS). The next day, plates were washed three times with PBST and incubated with 100 µL of diluted antibodies for 1 hour at RT. All the antibodies were diluted in MTPBS. After 1 hour incubation, wells were washed again three times, and 100 µL of horseradish peroxidase (HRP)-conjugated donkey anti-human IgG (FC specific; Jackson ImmunoResearch Laboratories, West Grove, PA) prediluted 1:5,000 in MTPBS were added to each well. The plate was incubated for 1 hour at RT, wells were then washed three times with TPBS, and 100 µL of TMB substrate solution (300 µL of 4 mg/mL 3,3,5,5-tetramethylbenzidine (MilliporeSigma) in DMSO, 10 mL of 0.1 M sodium acetate, pH 5.0, and 5 µL of fresh 30% hydrogen peroxide) were added to each well. After ~1 hour incubation at RT, the HRP reaction was stopped by adding 50 µL of 0.5 M H_2_SO_4,_ and optical density at 450 nm was measured using Synergy|H1 microplate reader (BioTek).

### Western blot

The culture supernatant containing AD8 gp120 or purified protein was separated on 8%–16% SDS-PAGE gel (mini-PROTEAN TGX protein gels; Bio-Rad) and transferred to a 0.45 µm nitrocellulose membrane (cat # 1620115, Bio-Rad). The membrane was blocked with 5% blotting-grade dry skim milk (cat # 1706404, Bio-Rad) in PBS (5%MPBS) overnight at 4°C. The next day, the membrane was washed with PBS and incubated for 1 hour on a shaker with serum of a person living with HIV-1 (1:10,000 dilution) and sheep anti-gp120 IgG (1:4,000 dilution; cat # 288, NIH AIDS reagent program), both diluted in 5%MPBS. After three washes with 0.05% Tween 20 (cat # 1706531, Bio-Rad) in PBS (TPBS), the membrane was incubated with peroxidase-conjugated anti-human IgG (1:10,000 dilution) and anti-sheep IgG (1:10,000 dilution) (Jackson ImmunoResearch Laboratories) in 5%MPBS for 1 hour. The membrane was washed three times with TPBS, developed with SuperSignal West Pico PLUS Chemiluminescent Substrate (cat # 34580, ThermoFisher Scientific), and analyzed using the Odyssey imaging system (LI-COR Biosciences). In some cases, we used serum from a person living with HIV-1 (1:10,000 dilution) without sheep anti-gp120 IgG.

### Viral assay

Viral neutralization assay in the presence and absence of soluble purified AD8 gp120 was performed as previously described (81).

## RESULTS AND DISCUSSION

### Generation and selection of stable HEK 293-AD8gp120 cells

**Fig 1:**
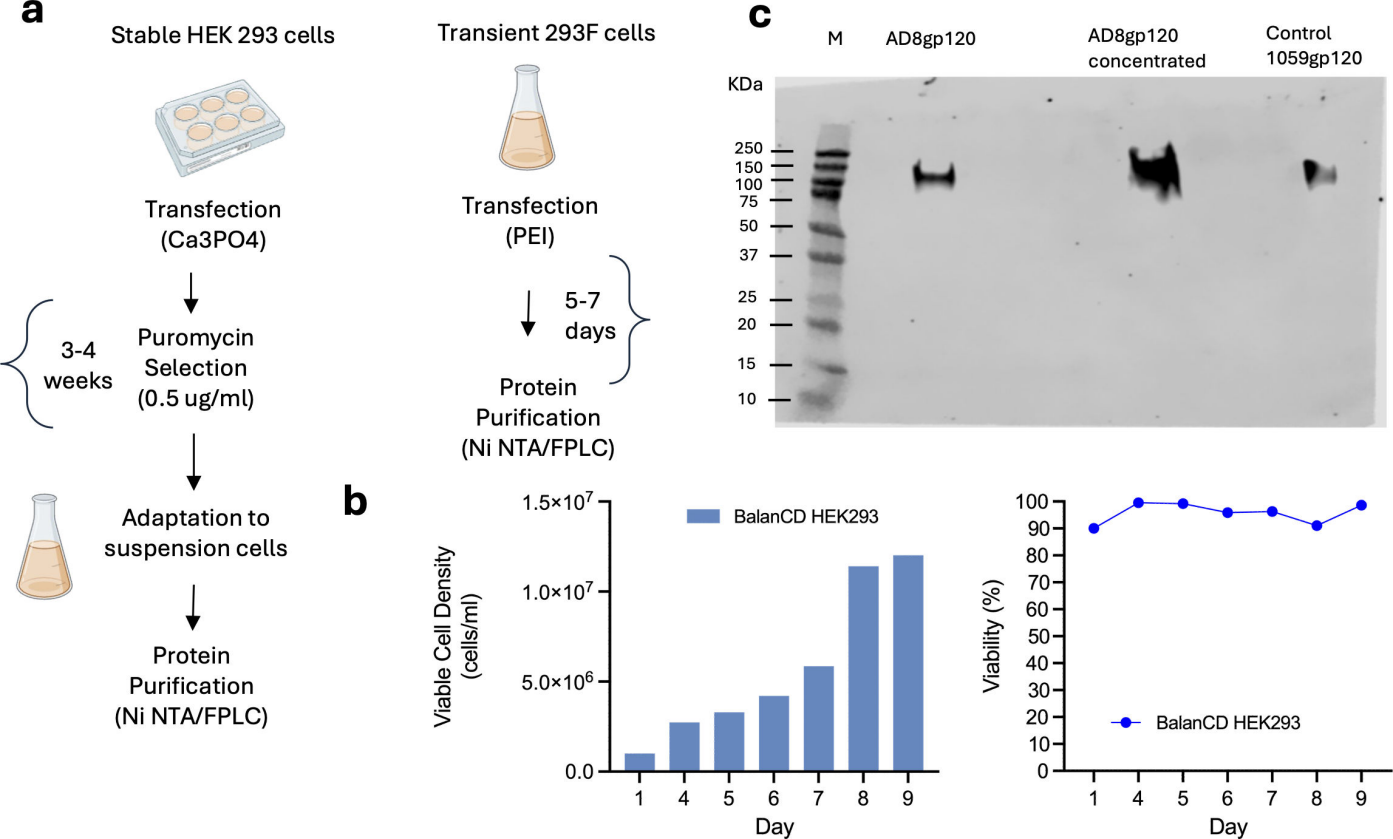
Generation of stable HEK293-AD8gp120 cells. (**a**) Experimental setup of stable and transient gene expression strategies of 293 cells. (**b**) Cell density and viability of stable HEK293-AD8gp120 cells after adaptation in BalanCD HEK293 medium. (**c**) Western blot analysis of AD8 gp120 expression in the supernatant of stable HEK293-AD8gp120 cells 48 hours after puromycin selection. The sample was concentrated using 30 kDa Vivaspin tubes; purified 1059-gp120 was used as the positive control.

HEK293 cells, originally derived from human embryonic kidney tissue, are widely used for protein expression due to high transfection efficiency of DNA into these cells by most available methods. We transfected adherent HEK293 cells with a plasmid encoding for HIV-1_AD8_ gp120 and the puromycin resistance gene by calcium phosphate (81). To generate a stable cell line expressing AD8gp120, transfected cells were selected with 0.5 µg/mL puromycin starting 24 hours post-transfection and selection continued for 3–4 weeks, with medium changes every 2–3 days to remove dead cells (Fig. 1). We transfected and selected adherent HEK293 cells rather than using HEK293 that have been already adapted for growth in suspension because adherent cells that die are readily removed with the medium, allowing us to maintain only cells that resist puromycin in culture. The cell culture supernatant was analyzed 72 hours post-transfection by Western blot, confirming the correct size of the expressed gp120 protein (Fig. 1c).

### Adaptation of HEK293-AD8gp120 to grow in suspension

Stable, adherent HEK293 cells expressing AD8 gp120 were next adapted to grow in suspension. In preliminary experiments, we tested media procured from different suppliers including Ex-Cell (MilliporeSigma), HEK GM (Sartorius), and BalanCD HEK293 (Irvine Scientific) and chose the latter. HEK293-AD8gp120 cells were then detached, washed, and suspended at an initial cell density of 1 × 10^6^ cells/mL in a total volume of 20 mL BalanCD HEK293. Suspension cultures of HEK293 were grown in shaker flasks in the presence of 0.5 µg/mL puromycin, and cell density and viability of the stable HEK293-AD8gp120 cells were monitored daily. We observed a steady increase in viable cell density and successful adaptation to suspension. Notably, when supplemented with 4% feed every other day to maintain optimal growth and productivity, the cells grew to a density of >1 x 10^7^ cells/ml with ~90% viability after 9 days in culture.

**Fig 2:**
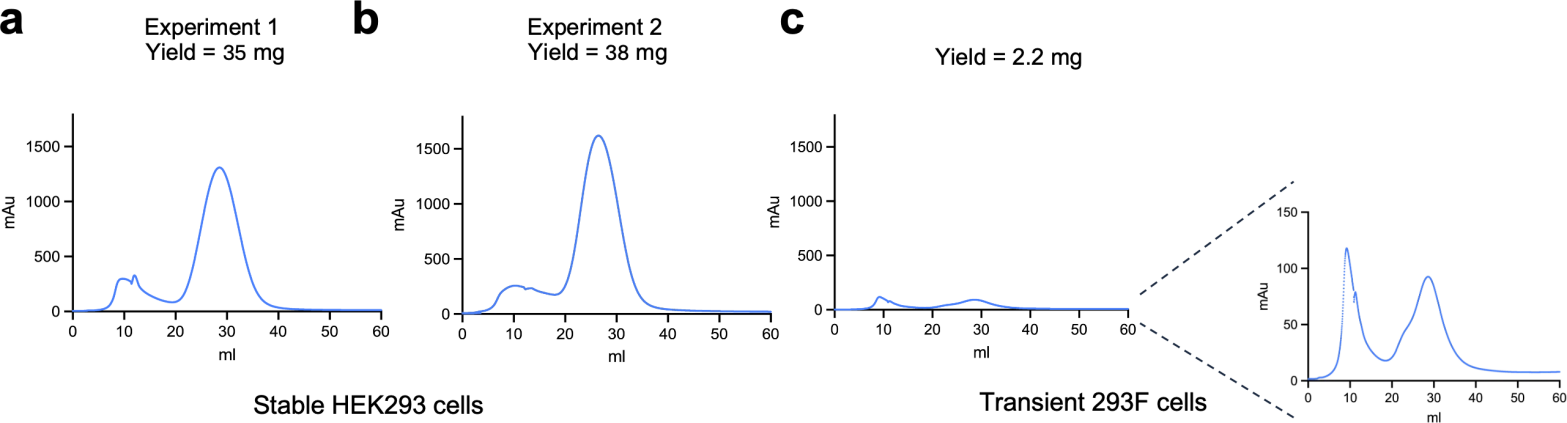
Purification of AD8gp120. Fast protein liquid chromatography (FPLC) chromatograms of AD8 gp120 purification from stable HEK293-AD8gp120 (**a** and **b**; each from 240 mL culture) and FreeStyle 293 F cells transiently transfected (**c**; 1 L). All volumes shown start from when an increase of mAu was detected.

**Fig 3:**
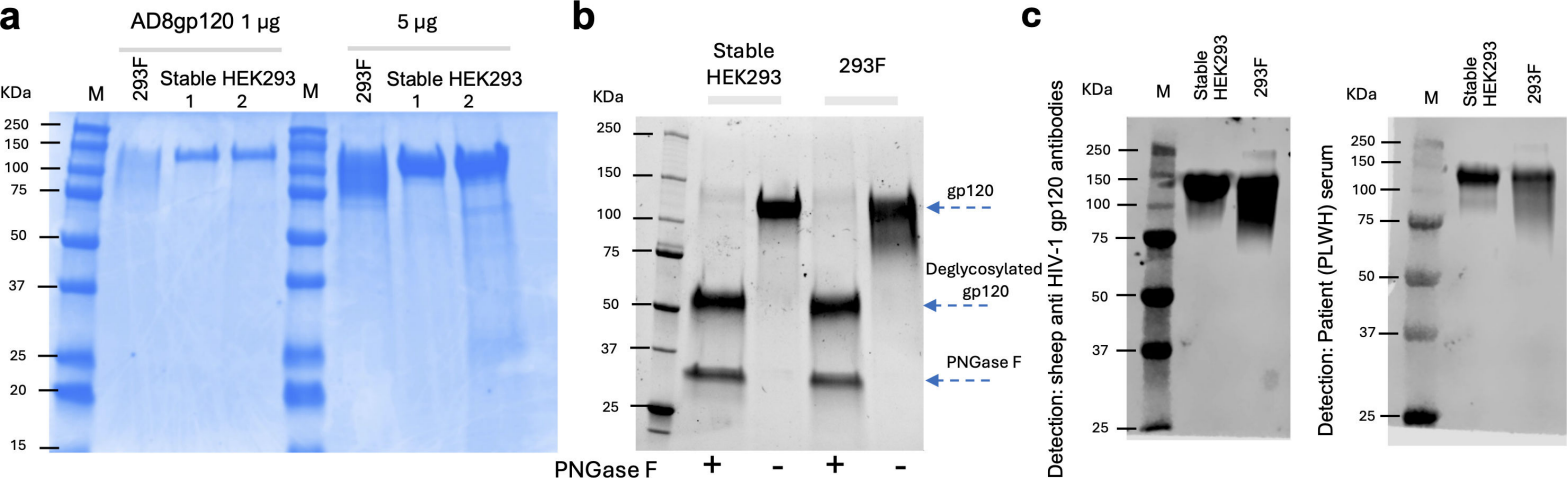
Purified AD8 gp120. (**a**) SDS-PAGE analysis of AD8 gp120 purified from the supernatant of PEI-transfected FreeStyle 293 F cells and stable HEK293-AD8gp120 cells. Protein samples (1 µg and 5 µg) were analyzed. (**b**) Glycosylation analysis of purified gp120 from the two preparations; an extended spread of gp120 purified from FreeStyle 293 F likely reflects hyper-glycosylation, and both gp120s show similar size and homogeneity after deglycosylation by PNGase F. (**c**) Western blot analysis of the purified gp120s using different antibodies for detection.

As reference/control, we used PEI to transiently transfect FreeStyle 293 F cells, with the same AD8gp120 expression plasmid used to generate the stable HEK293 cells. We used PEI for transient transfection and not calcium phosphate because, in our experience, 293 cells in suspension are more efficiently transfected with PEI, whereas adherent 293 cells are more efficiently transfected with calcium phosphate. Thus, we used optimized transfection reagent for each case. In addition, as the transfection efficiency increases, stable expression in cell populations may reflect more diverse integration sites (more integration events are potentially possible). However, once cells are selected, most cells are expected to express soluble AD8 gp120 with levels that depend on the specific integration site and cellular and external environments. FreeStyle 293 F transfected cells were grown in the recommended medium for 7 days. Supernatants of cells transiently or stably expressing AD8 gp120 were harvested by centrifugation and then equilibrated overnight against binding buffer. gp120 was purified from the supernatants by liquid chromatography system (AKTA Go; Cytiva) using 5 mL Ni-NTA column. We observed a few differences between the elution profiles of gp120 from the two sources of supernatants. The chromatogram of eluted gp120 from the stable HEK293-AD8gp120 supernatant showed sharp peaks with good separation and a dominant peak corresponding to gp120 according to subsequent SDS-PAGE and Western blot confirmation analysis (Fig. 2 and 3). In contrast, the chromatogram of eluted gp120 from transient FreeStyle 293 F cell supernatants showed two peaks of comparable size, and the overall yield was ~50 fold lower compared to the stable expression (Fig. 2; Table 1). Purity of gp120 was evaluated by SDS-PAGE analysis, which showed a single band of the correct ~120 kDa size for both preparations (Fig. 3). However, gp120 purified from the stable HEK293 cells exhibited higher homogeneity compared to the gp120 purified from FreeStyle 293 F cells. Western blot analysis using either sheep polyclonal antibodies against gp120 or serum from a PLWH further confirmed the difference between the two preparations observed by the SDS-PAGE analysis. To test the contribution of glycans to gp120 homogeneity, we used PNGase F to actively remove the glycans from the purified gp120s. The PNGase F enzyme specifically recognized and removed N-linked glycans. Both PNGase-F-treated samples showed the same protein size and homogeneity after the removal of glycans, according to SDS-PAGE analysis. Thus, stable expression of AD8 gp120 was associated with more homogenous processing or modifications, most likely glycosylation, due to a more controlled environment during protein expression, lower copy number of the gp120 gene in each cell, and the slower rate of growth in comparison to the FreeStyle 293 F cells.

**TABLE 1:** Experimental conditions and yield of gp120 production

Cells	Virus	Exp.^*a*^	Transfection	Expression	Culture	Yield (mg/L)	Conditions	Culture time
gp120								
FreeStyle 293 F	HIV-1_AD8_	1	PEI	Transient	1 L	~ 2.2	125 RPM 37°C 8% CO_2_ FPLC purification	7 days; 50% media added 1 day post-transfection
FreeStyle 293 F	HIV-1_AD8_	2	Turbo	Transient	1 L	~ 4	125 RPM 37°C 8% CO_2_ gravity flow purification	5 days
HEK293	HIV-1_AD8_	1	Calcium phosphate	Stable	200 mL + 40 mL feed	~ 148	140 RPM 37°C 8% CO_2_ FPLC purification	7 days + 4% feed every other day for 10 days
HEK293	HIV-1_AD8_	2	Calcium phosphate	Stable	200 mL + 40 mL feed	~ 162	140 RPM 37°C 8% CO_2_ FPLC purification	7 days + 4% feed every other day for 10 days + 3 days
FreeStyle 293 F	HIV-1_1059_	1	Turbo	Transient	1 L	~ 6	125 RPM 37°C 8% CO_2_ gravity flow purification	5 days
HEK293	HIV-1_1059_	1	Calcium phosphate	Stable	200 mL + 40 mL feed	~ 50	140 RPM 37°C 8% CO_2_ FPLC purification	9 days + 4% feed every other day for 10 days
SOSIP								
FreeStyle 293 F	HIV-1_1059_	1	PEI	Transient	1.5 L	~0.25^*b*^	125 RPM 37°C 8% CO_2_ FPLC purification	6 days
HEK293	HIV-1_1059_	1	PEI	Transient	400 mL + 40 mL feed	~2.7^*b*^	125 RPM 37°C 8% CO_2_ FPLC purification	10 days with 4 additions of 4% feed every other day

^*a*^ Exp., experiment number.

^*b*^ Yield of purified 1059-SOSIP was calculated from the FPLC chromatograms using the theoretical extinction coefficient 1.61 for 1059-SOSIP.

### HIV-1 AD8 gp120 antigenicity

**Fig 4:**
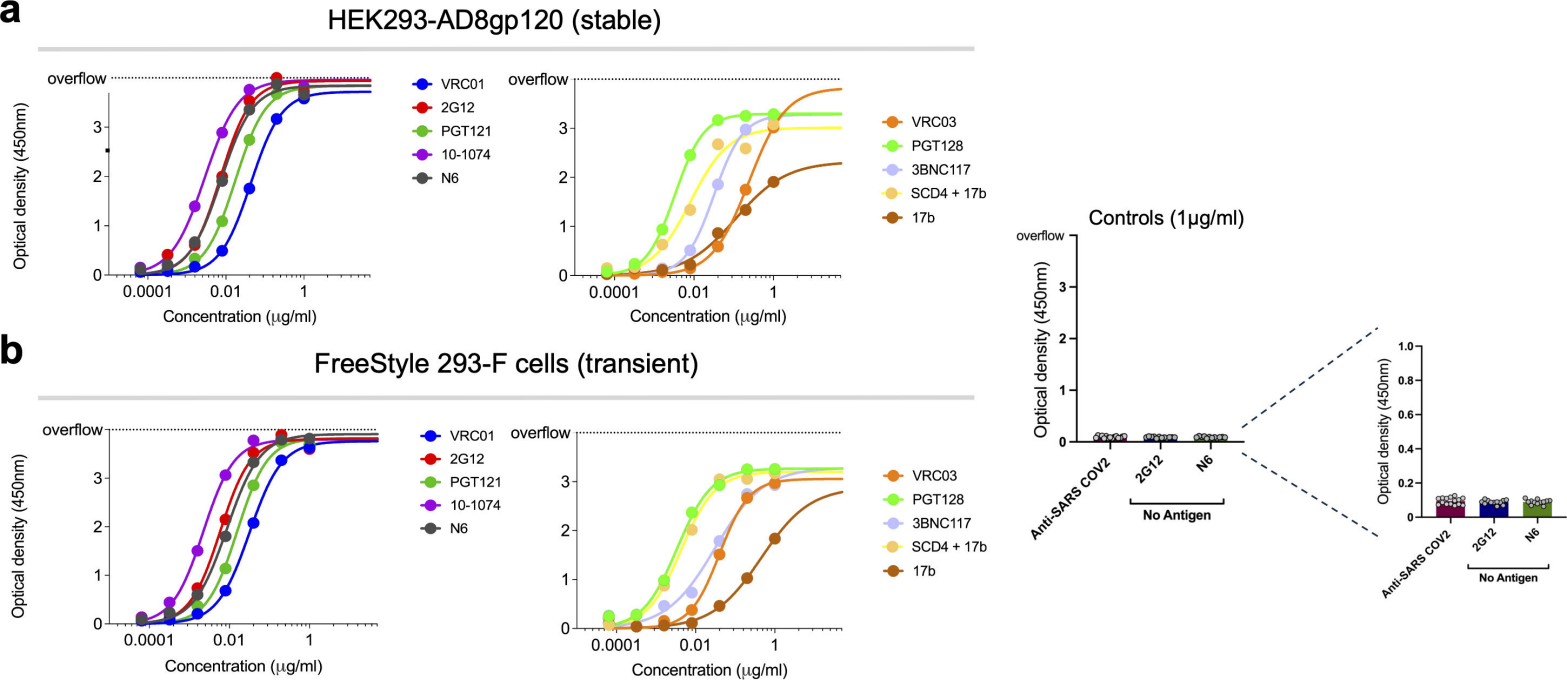
Binding of purified HIV-1 AD8 gp120 to antibodies assessed by ELISA. Binding of AD8 gp120 purified from stable HEK293-AD8gp120 cells (**a**) and from transiently transfected FreeStyle 293 F (**b**) to different antibodies. Controls include anti-SARS-CoV-2 spike antibody and wells with no antigen and are shown on the right.

We compared the antigenic profile of the monomeric gp120 purified from the 2 cell sources by ELISA. We used the following antibodies: VRC01, VRC03, 3BNC117, N6, 2G12, PGT121, 10-1074, PGT128, and 17b (with or without soluble CD4) and a control antibody against the SARS-CoV2 spike. These antibodies target the CD4-binding site (VRC01, VRC03, 3BNC117, and N6) (90–93), the glycans at the base of gp120 V3 loop (2G12, PGT121, 10-1074, and PGT128) (94–96), and the CD4-induced site, which overlaps with the coreceptor binding site (17b) (97). The antibodies were tested at a wide range of concentrations between 0.0001 and 1 µg/mL, and all showed high binding to gp120. Binding of all antibodies showed a typical dose response and, except for 17b, reached saturation at 1 µg/mL antibody concentration. The addition of sCD4 increased the 17b binding to gp120 to similar levels of the binding of the other antibodies. For all antibodies, binding to the monomeric gp120 purified from two different cell sources was comparable, suggesting no significant difference in their antigenicity. As expected, binding of anti-SARS-CoV-2 spike to gp120 as well as binding of the anti-Env antibodies in the absence of gp120 was comparable to the background (Fig. 4).

### HIV-1 AD8 gp120 function

**Fig 5:**
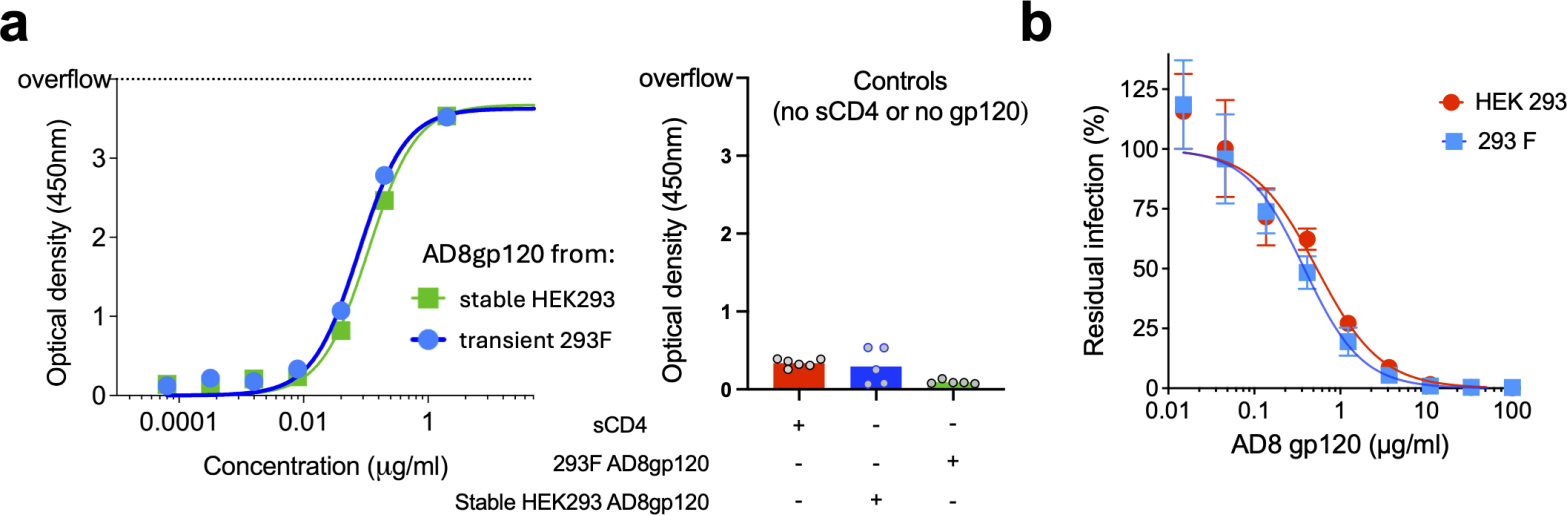
Function of purified AD8 gp120. (**a**) Dose-response curve of binding of AD8 gp120 purified from stable HEK293-AD8gp120 cells and from transiently transfected FreeStyle 293 F to immobilized sCD4. (**b**) Effects of purified AD8 gp120 on viral entry by viruses pseudotyped with HIV-1_AD8_ Envs.

We next assessed the function of purified AD8 gp120 from the two cell sources. We first measured the binding of soluble gp120 to immobilized sCD4 by ELISA. The gp120 from both sources exhibited a typical dose-response curve that reached a saturation at 1 µg/mL gp120 concentration. No significant difference between the two preparations was observed, and controls without either sCD4 or gp120 showed very low nonspecific binding (Fig. 5). We then measured the ability of gp120 to compete with and block viral infection by viruses pseudotyped with HIV-1 AD8 Envs (gp160). The response to soluble gp120 from both cell sources exhibited similar patterns of neutralization, with an IC50 value of approximately 0.5 µg/mL. Thus, the gp120 function of the monomeric protein from two preparations was consistent with known biological activities of gp120 and exhibited the expected and very similar phenotype in both cases.

### Production of HIV-1 1059-SOSIP trimer

**Fig 6:**
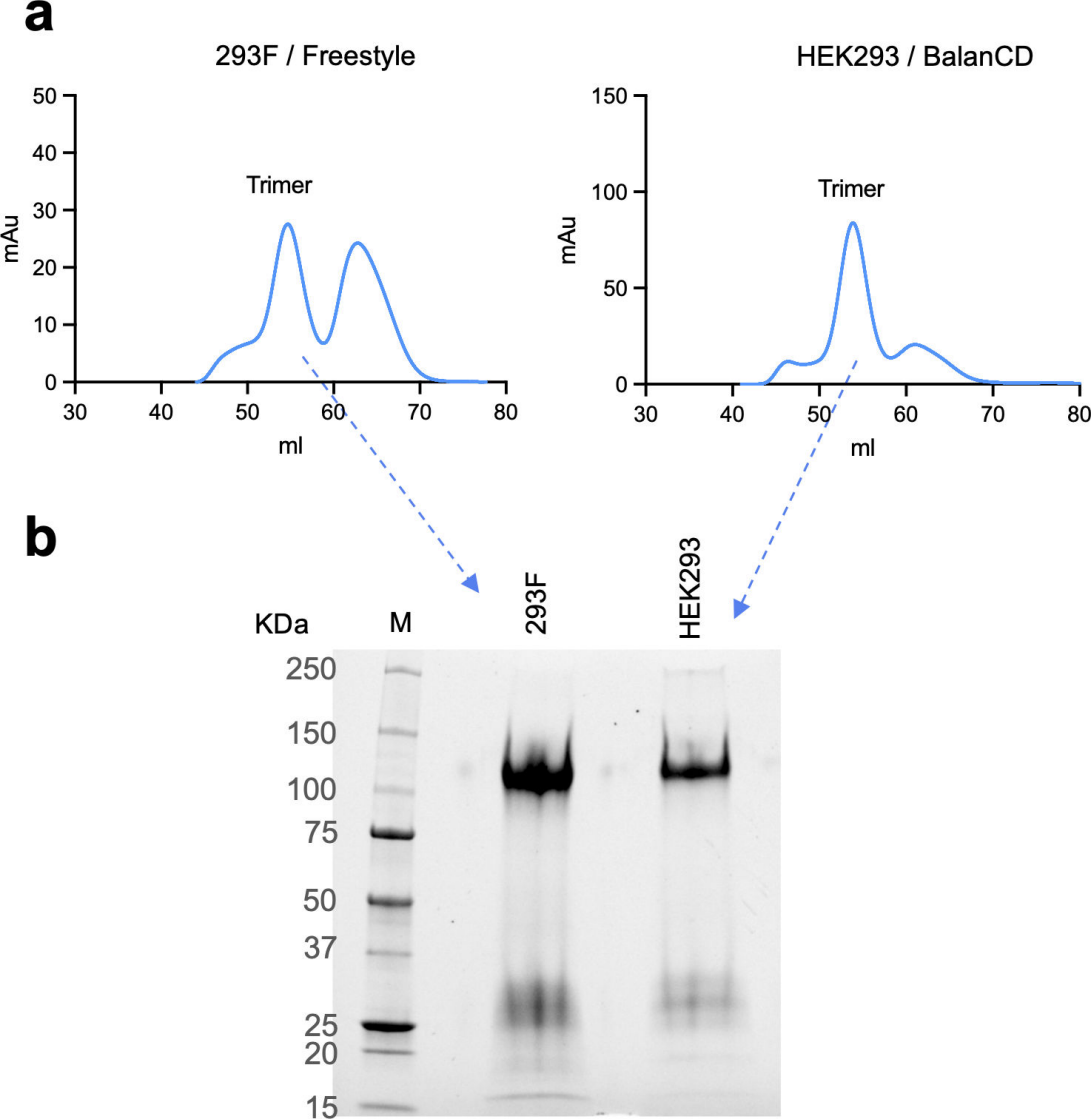
Production of trimeric 1059-SOSIP Envs. (**a**) FPLC chromatograms of 1059-SOSIP Env purification from FreeStyle 293 F cells or HEK293 grown in BalanCD medium, both transiently transfected using PEI. (**b**) SDS-PAGE analysis of trimeric fractions of purified 1059-SOSIP proteins.

As many studies now use the trimeric form of soluble HIV-1 Envs for different applications and experiments, we next studied the production of soluble trimeric 1059-SOSIP Envs. The 1059 Envs have been used for DNA-based vaccination of humans (98), and 1059 gp140 has been used to measure antigen binding of serum from nonhuman primates vaccinated with DNA-based 1059 immunogen (99). Thus, the 1059-SOSIP trimer serves as a good example for trimer production. We transfected both 293 F in FreeStyle medium and HEK293 in BalanCD medium with the same plasmid supporting 1059-SOSIP expression. Both cells were grown in suspension and transfected with PEI. We purified the 1059-SOSIP trimer from the supernatant of cells using Galactose Nivalis lectin column, followed by size exclusion chromatography. Consistent with our results for the gp120 production, we detected a significantly higher yield of 1059-SOSIP that was produced in HEK293 grown in BalanCD medium (with feed) compared to the production in 293 F cells grown in FreeStyle medium. Notably, the higher fraction of trimeric protein was formed in HEK293 cells compared to the 293 F cells (Fig. 6)

Here, we report the efficient production of HIV-1 AD8 gp120 from stable HEK293 cells grown in suspension. Establishment of the HEK293-AD8gp120 cells was straightforward and required only a few weeks of selection; even without further selection for single clones, we could purify hundreds of milligrams from a liter of cells grown in suspension, which was significantly higher (approximately 50-fold higher; Table 1) than the yield of FreeStyle 293 F cells transiently transfected with the same gp120 expression plasmid. The stable HEK293-AD8 gp120 cells grew in suspension at a slower rate than the parental adherent 293 grown in DMEM that included serum; however, the stable HEK293-AD8 gp120 cells grew to very high density of >1 × 10^7^ cells/mL in suspension. Thus, the high number of stable cells produced significantly higher amounts of proteins than transient transfection. Moreover, the slower and more controlled growth of the HEK293 in BalanCD medium in comparison with the 293 F cells led to more homogenous glycosylation patterns, which may be important for presentation to the immune system and for biological activity. We obtained similar results for production of HIV-1 gp120 of a second HIV-1 strain (1059) and about 10-fold higher yield for the stable HEK293-1059gp120 compared to the transient transfection of the same plasmid into FreeStyle 293 F cells (Table 1). Additionally, we obtained similar results with a different soluble format (SOSIP) of HIV-1 Envs. 1059 Envs have been used for DNA-based vaccination of humans (98), and 1059-SOSIP could be efficiently produced in HEK293 cells grown in suspension in BalanCD medium. Efficient 1059-SOSIP production was associated with increased yield and higher fraction of trimeric 1059-SOSIP protein compared to 1059-SOSIP production in 293 F cells. Thus, at least for two different gp120s and 1 SOSIP Envs, we observed highly efficient production of soluble HIV-1 Envs. The method is appropriate for laboratories that may need high amounts of proteins for different experiments.

## Data Availability

All data are available in the article.

## SUPPLEMENTAL MATERIAL

Text S1 jvi.01359-25-s0001.txt pAD8gp120 sequence.

Text S2 jvi.01359-25-s0002.txt pTPA-1059-SOSIP sequence.

ASM does not own the copyrights to Supplemental Material that may be linked to, or accessed through, an article. The authors have granted ASM a non-exclusive, world-wide license to publish the Supplemental Material files. Please contact the corresponding author directly for reuse.
